# Guideline review: 2025 British Society of Gastroenterology guidelines on inflammatory bowel disease in adults – Part 2: ulcerative colitis

**DOI:** 10.1136/flgastro-2025-103426

**Published:** 2025-11-25

**Authors:** Thomas D Butler, Jonathan P Segal

**Affiliations:** 1 Division of Diabetes, Endocrinology and Gastroenterology, Faculty of Biology, Medicine and Health, The University of Manchester, Manchester, M13 9PT, UK; 2 Northern Care Alliance NHS Foundation Trust, Salford, Manchester, UK; 3 Department of Gastroenterology, Royal Melbourne Hospital, Parkville, Victoria, Australia; 4 Department of Medicine, University of Melbourne, Parkville, Victoria, Australia

## Abstract

This guideline review summarises the key recommendations for the diagnosis and management of ulcerative colitis from the updated 2025 British Society of Gastroenterology guidelines on inflammatory bowel disease in adults.

## Introduction

Ulcerative colitis (UC) is a chronic inflammatory condition presenting with symptoms including diarrhoea, rectal bleeding and abdominal pain. UC can involve a few centimetres of distal rectum through to the whole colon. The spectrum of severity is broad, with the most severely affected patients exposed to multiple medical treatments and/or colectomy. Acute severe UC still carries a risk of mortality. Since the 2019 British Society of Gastroenterology (BSG) guidelines, there have been significant advances in therapeutic options for UC. This article summarises the key recommendations for the management of UC included in the 2025 BSG inflammatory bowel disease (IBD) Guidelines^1^.

## Methodology

The guideline update prioritises key thematic questions agreed prospectively through a Delphi process. Recommendations follow a robust GRADE methodology, as described in the guideline protocol ^2^. An annotated figure and table of definitions are included within the guideline for readers unfamiliar with the specifics of GRADE language.

## Diagnosis of ulcerative colitis

The diagnosis and monitoring of UC requires a thorough history, examination, and correlation with clinical scores, blood tests, faecal calprotectin, imaging, endoscopy and histology. In the absence of acute severe UC (discussed later), colonoscopy with segmental biopsy is the recommended test for diagnosis and assessment of disease extent. Infections (including sexually transmitted infections) and medications such as non-steroidal anti-inflammatories should be considered as differential precipitants of colitis with elevated inflammatory biomarkers, and the guidelines emphasise the importance of providing adequate clinical information for histopathology assessment.

## Monitoring in ulcerative colitis

Clinical scoring systems are frequently used in UC clinical trials, but not yet regularly incorporated into clinical practice. Patient reported outcome measures (PROMs) such as PRO-2 and IBD Control hold potential for identifying unmet needs and personalised care^3^, but the guidelines do not elaborate on their use, leaving an opportunity for local research networks to assess and validate PROMs.

Faecal calprotectin should be used as a quantitative, non-invasive marker of inflammation to monitor response to treatment. The guidelines take a pragmatic approach, suggesting a faecal calprotectin <100μg/g could represent endoscopic remission and values >200μg/g should warrant consideration of re-assessment with colonoscopy. It is also important to consider an individual’s trend in faecal calprotectin.

Due to the mucosal, non-penetrating nature of UC, cross-sectional imaging plays less of a role in monitoring compared with CD. However, a section on intestinal ultrasound (IUS) is presented as a non-invasive and patient-friendly approach to monitoring of active disease in UC. The emergence of IUS scoring systems that incorporate factors such as bowel wall thickness and doppler flow aim to standardise IUS results, demonstrating good correlation with endoscopic disease activity. However, access to IUS is still limited and anticipated expansion of IUS services across the UK must continue to employ this evidence in daily practice.

Treatment targets are still a matter for debate and the guidelines suggest a multimodal approach for patient monitoring and a treat-to-target approach as laid out by STRIDE II ^4^. Whilst there is growing evidence that mucosal healing, defined by endoscopic remission (e.g. Mayo endoscopic subscore = 0 or UCEIS <= 1) is associated with better long-term outcomes, the service implications of reaching mucosal healing are not yet clear, including costs of increased endoscopic surveillance, and risks associated with lower thresholds for switching therapy in pursuit of mucosal healing. The IBD community needs to continue to define optimal treatment targets, which are attainable across the UK with appropriate resource utilisation.

Patients with IBD require serial endoscopic screening, given their higher risk of colorectal cancer particularly in those with chronic, active, extensive inflammation. The IBD surveillance strategy has recently been updated in a separate BSG guideline^5^.

## Management of ulcerative colitis

### Mild to moderate ulcerative colitis

There are minimal changes to the management of mild to moderate UC. 5-aminosalicylic acid (5-ASA) therapy is recommended for the induction and maintenance of mild to moderate UC, due to the high certainty of evidence that 5-ASAs have a moderate magnitude of effect. Oral corticosteroids are recommended following 5-ASA failure. If remission is achieved with a course of corticosteroids, 5-ASA maintenance therapy can be reconsidered.

### Moderate to severe ulcerative colitis

Oral corticosteroids are also recommended for induction of remission in moderate to severe UC. In contrast to Crohn’s disease, the guidelines do not recommend early initiation of advanced therapy in patients with moderate to severe UC, and purine analogues such as azathioprine monotherapy can be used for maintenance of remission in UC (but not induction of remission). The guidelines impress the importance of avoiding multiple and prolonged corticosteroid courses, which are associated with significant harm and should be used sparingly, given the burgeoning availability of therapeutic agents. Therefore, initiation of advanced therapy is recommended where there is inadequate response within two weeks of oral corticosteroid therapy, an unsuccessful oral corticosteroid taper, or to avoid repeated corticosteroid courses.

Since the introduction of infliximab, advanced therapies have revolutionised IBD treatment by improving patient quality of life, clinical outcomes and reducing steroid use. In UC, advanced therapies recommended by the previous guideline included infliximab, adalimumab, golimumab, vedolizumab and tofacitinib. The current guideline contains six novel advanced therapies for the induction and maintenance of moderate to severe UC, in addition to ustekinumab, which received NICE approval shortly after previous guideline publication (Table 1). The novel therapies target an array of immune molecules including the JAK-STAT pathway (upadacitinib, filgotinib), IL-23 (risankizumab, mirikizumab) and the sphingosine-1-phosphate receptor (S1PR) (etrasimod, ozanimod). This poses a positive challenge for treatment selection, which should remain committed to principles of considering patient and disease factors, previous treatments and local availability, all via a multi-disciplinary approach.

The guideline does not provide a hierarchy of treatments, due predominantly to the lack of head-to-head data, but network meta-analysis (NMA) of trials for induction and maintenance of moderate to severe UC demonstrate a large magnitude of effect for upadacitinib and tofacitinib, with moderate magnitude of effect for risankizumab, filgotinib and ozanimod, and a small magnitude of effect for infliximab, etrasimod, golimumab, mirikizumab, ustekinumab and vedolizumab. The guidelines recommend upadacitinib for induction and maintenance of moderate to severe UC, while suggesting the use of other listed advanced therapies (Table 1). According to GRADE terminology, ‘suggest’ is a conditional statement, and ‘recommend’ is a strong statement reflecting the high certainty of evidence that upadacitinib has a large magnitude of effect. However, of note, the strength of recommendation for Upadicitinib in UC is still regarded as conditional in the guideline, due to the current lack of long-term safety data. This terminology reflects the ambiguity that can still appear when methodological rigour meets clinical practice. The strength of a GRADE statement does not necessarily represent a magnitude of effect or a treatment hierarchy, and treatment choices should be individualised rather than protocolised. The guideline provides a helpful table (table 7 in ref ^1^) of estimated time to treatment goals for each therapy in UC.

### Adalimumab is not suggested for the induction and maintenance of remission in patients with moderate to severe ulcerative colitis

Notably, in a departure from 2019 guidelines, adalimumab is not suggested for induction and maintenance of remission in moderate to severe UC. This is driven by the results of the NMA, which for adalimumab included 1917 patients across four randomised controlled trials and demonstrated a trivial magnitude of effect for clinical response, clinical remission and endoscopic response. The definitions for magnitude of effect were pre-determined by the guideline development group ^2^. The safety profile of adalimumab remains low risk. Given the availability of more efficacious advanced therapies, adalimumab is no longer suggested as a standard advanced therapy in UC. This will require careful interpretation by IBD teams and adalimumab therapy should not be stopped without shared decision-making.

Adalimumab may remain a suitable therapy in people with extra-intestinal manifestations or concurrent immune-mediated inflammatory diseases. Of note, the overall certainty of evidence is low and as with all conditional GRADE statements, further evidence could influence future statements.

### Other therapeutic strategies in ulcerative colitis

Currently, antibiotics, probiotics and faecal microbial transplant are not suggested for the induction or maintenance of remission in UC, due to insufficient evidence of efficacy and/or safety.

Treatment withdrawal should generally be approached with caution in IBD. 5-ASAs can be discontinued if UC patients achieve prolonged remission with advanced therapy, but the chemo-preventative effect of 5-ASAs in combination with advanced therapy is unknown. Withdrawal of purine analogues as either monotherapy or combination therapy is associated with a risk of relapse, which must be weighed against the long-term risks of lymphoproliferative disorders and cancers of the skin and urinary tract. Even in patients with prolonged mucosal healing, withdrawal of advanced therapy is associated with a 50% risk of relapse in the first year ^6^, and is not recommended by the guidelines.

### Acute severe ulcerative colitis

Acute severe ulcerative colitis (ASUC) is defined by Truelove and Witts’ criteria. Despite medical advances, there is still a mortality risk with ASUC and roughly 20% of ASUC patients will require colectomy during admission. Initial management is unchanged in this guideline, including urgent assessment of vital observations, blood tests (including early pre-biologic screen) and stool culture including *Clostridium difficile* assay. Abdominal imaging is often required, and CT scan is preferred over abdominal X-ray if perforation or intra-abdominal collection is suspected. Within 24 hours of admission, flexible sigmoidoscopy, often without bowel preparation, is essential to confirm diagnosis, assess severity and obtain tissue for histological examination and *Cytomegalovirus* evaluation. Intravenous (IV) corticosteroids should be started without delay, alongside supportive measures and venous thromboembolic prophylaxis.

Rescue therapy with ciclosporin or infliximab is indicated if there is lack of response to IV corticosteroids on day 3, objectively scored using tools such as the Travis criteria ^7^. In this guideline, an intensified infliximab dosing regimen can be considered in selected groups of steroid-refractory ASUC patients, particularly if albumin level is low. However, data are conflicting on the benefits of this strategy and PREDICT-UC, the only randomised trial in this area did not demonstrate a difference in short- and long-term outcomes ^8^.

In light of the off-label reports of successful JAK inhibitor use in ASUC, combined with interim analysis from a phase 4 trial in Canada (NCT04925973), JAK inhibitor use as rescue therapy in ASUC can be considered in a highly selected, steroid-refractory group where conventional rescue therapy and surgery is contraindicated. Surgical subtotal colectomy and ileostomy is still recommended for patients who have not responded to rescue medical therapy and the colorectal surgical team should be involved from an early stage of admission.

### Proctitis

Proctitis is UC limited to the rectum, extending up to 15-20cm from the anal verge. Whilst limited in extent, it can be severe in activity with a non-trivial colectomy rate. Topical 5-ASA therapy is effective for induction and maintenance of mild or moderately active proctitis. Non-responders can be treated with oral 5-ASA therapy or rectal topical corticosteroid therapy. Refractory proctitis is managed in line with moderate to severe UC outlined above.

### Pouchitis

UC patients undergoing ileal pouch-anal anastomosis following proctocolectomy have a high risk of developing pouchitis. Ongoing symptoms following pouch formation surgery can be investigated with pelvic MRI scan, stool culture and pouchoscopy. Consistent with prior guidelines, acute pouchitis can be treated with a 2-week course of antibiotics (such as ciprofloxacin or metronidazole) and may require repeated courses. Patients should be counselled on antibiotic side effects. Advanced therapies for the treatment of antibiotic-refractory chronic pouchitis have traditionally been extrapolated from UC treatment algorithms given the paucity of good quality data. Results from the EARNEST trial enable the recommendation for vedolizumab as first line advanced therapy in chronic pouchitis ^9^, although longer term data are still required.

### Pregnancy in IBD

For most women, a diagnosis of IBD will not impact their fertility. In addition to general health measures including vitamin D and folic acid supplementation, an individualized management plan during pregnancy is recommended. Well controlled IBD is the primary aim, which includes good patient education, planning of IBD care prior to pregnancy where possible and safe medical therapy where required. Venous thromboembolic prophylaxis should be considered for hospitalised patients, outpatients with active IBD during the third trimester, and following caesarean section.

Active IBD in pregnancy carries higher risk of adverse outcomes including low birth weight, spontaneous abortions and stillbirths ^10^. Therefore, active IBD in pregnancy requires treatment. Corticosteroids can be prescribed when essential, with close monitoring of complications such as gestational diabetes. Anti-TNF therapy, vedolizumab and ustekinumab are considered safe, and guidelines recommend continuation of these advanced therapies throughout pregnancy and breast feeding. Initiation of purine analogues should be avoided in pregnancy, due to the risk of immune-mediated pancreatitis. Medical treatments contraindicated in pregnancy include methotrexate, upadacitinib, filgotinib, tofacitinib, etrasimod and ozanimod, which should be stopped 3 months prior to conception, where possible. The guidelines do not provide a recommendation on newer anti-IL-23p19 therapies in pregnancy.

## Future directions

The significant advancement in treatment options since publication of the 2019 guidelines demonstrates the fast pace of change within IBD. Rapid developments in IBD management risk outpacing future *de novo* guideline development. Living guidelines, as employed by the American Gastroenterological Association (AGA) ^11^, allow for updates of individual recommendations as new data emerges. The extensive groundwork and NMAs undertaken by the methodologists for the new BSG IBD guidelines make this an attractive prospect.

## Conclusions

Management of UC continues to present a challenge for clinicians. The *BSG guidelines on IBD in adults* provides updated guidance on the diagnosis and management of UC. There is particular detail on the burgeoning number of available therapies to manage UC. This guideline will support clinicians in providing patient-centred, individualised and proactive management strategies for people living with IBD.

## Figures and Tables

**Table 1:** A summary of recommendations from the 2025 BSG IBD guidelines regarding the use of advanced therapies for induction and maintenance of remission in patients with moderate to severe IBD. Medications are not listed hierarchically. Abbreviations: UC, ulcerative colitis; CD, Crohn’s disease; TNF, tumour necrosis factor; S1PR, sphingosine-1-receptor; JAK, janus kinase; IL, interleukin.

Mechanism of action	Drug	Condition	Grade recommendation	Magnitude of effect	Certainty of evidence
Anti-TNF	Infliximab	UC	Suggested	Small	Moderate
		CD	Suggested	Moderate	Moderate
	Adalimumab	UC	Not suggested	Trivial	Low
		CD	Recommended	Moderate	Moderate
	Golimumab	UC	Suggested	Small	Low
S1PR modulator	Etrasimod	UC	Suggested	Small	Moderate
	Ozanimod	UC	Suggested	Moderate	Low
JAK inhibitor	Filgoti nib	UC	Suggested	Moderate	Low
	Upadacitinib	UC	Recommended	Large	High
		CD	Suggested	Small	Low
	Tofacitinib	UC	Suggested	Large	Moderate
Anti-IL-23	Mirikizumab	UC	Suggested	Small	Low
	Risankizumab	UC	Suggested	Moderate	Moderate
		CD	Suggested	Small	Low
Anti-IL-12/23	Ustekinumab	UC	Suggested	Small	Low
		CD	Suggested	Moderate	Moderate
Anti-integrin	Vedolizumab	UC	Suggested	Small	Moderate
		CD	Not suggested	Moderate	Low

## What is already known on this topic

Ulcerative colitis is an inflammatory bowel disease causing significant impact on quality of life. There are multiple considerations in the management of ulcerative colitis, which can be challenging for the patient, clinician and service provision.

## What this study adds

The *2025 BSG guideline on inflammatory bowel disease in adults* provides an update from the 2019 guidelines, reflecting recent advancements, particularly relating to novel therapeutic agents.

## How this study might affect research, practice or policy

This guideline provides clinicians with a comprehensive overview of the approach to diagnosis and management of people with inflammatory bowel disease. This review covers management of ulcerative colitis.
